# Defense Strategies for Asymmetric Networked Systems with Discrete Components

**DOI:** 10.3390/s18051421

**Published:** 2018-05-03

**Authors:** Nageswara S. V. Rao, Chris Y. T. Ma, Kjell Hausken, Fei He, David K. Y. Yau, Jun Zhuang

**Affiliations:** 1 Computational Sciences and Engineering Division, Oak Ridge National Laboratory, Oak Ridge, TN 37831, USA; 2 Hang Seng Management College, Hong Kong; chris.ytma@gmail.com; 3 Faculty of Social Sciences, University of Stavanger, 4036 Stavanger, Norway; kjell.hausken@uis.no; 4 Department of Mechanical and Industrial Engineering, Texas A&M University, Kingsville, TX 78363, USA; fei.he@tamuk.edu; 5 Information Systems Technology and Design Clusteer, Singapore University of Technology and Design, Singapore 487372, Singapore; david.ky.yau@gmail.com; 6 Department of Industrial and Systems Engineering, University at Buffalo, Buffalo, NY 14260, USA; jzhuang@buffalo.edu

**Keywords:** networked systems, cyber-physical infrastructures, aggregated correlation functions, sum-form, product-form and composite utility functions

## Abstract

We consider infrastructures consisting of a network of systems, each composed of discrete components. The network provides the vital connectivity between the systems and hence plays a critical, asymmetric role in the infrastructure operations. The individual components of the systems can be attacked by cyber and physical means and can be appropriately reinforced to withstand these attacks. We formulate the problem of ensuring the infrastructure performance as a game between an attacker and a provider, who choose the numbers of the components of the systems and network to attack and reinforce, respectively. The costs and benefits of attacks and reinforcements are characterized using the sum-form, product-form and composite utility functions, each composed of a survival probability term and a component cost term. We present a two-level characterization of the correlations within the infrastructure: (i) the aggregate failure correlation function specifies the infrastructure failure probability given the failure of an individual system or network, and (ii) the survival probabilities of the systems and network satisfy first-order differential conditions that capture the component-level correlations using multiplier functions. We derive Nash equilibrium conditions that provide expressions for individual system survival probabilities and also the expected infrastructure capacity specified by the total number of operational components. We apply these results to derive and analyze defense strategies for distributed cloud computing infrastructures using cyber-physical models.

## 1. Introduction

Infrastructures for cloud computing, science experiments and computations and smart energy grid consist of complex, geographically-dispersed systems that are connected over long-haul networks. In these infrastructures, the communications network plays a critical, asymmetric role of providing the vital connectivity between the systems such as cloud computing sites, or supercomputers, or energy distribution centers. Network failures render the individual systems unreachable and in extreme cases can render the entire infrastructure unavailable. Such an infrastructure is represented by its constituent systems, $S_{i}$, i=1,2,…,N, and the network connecting them is represented as a separate system $S_{N+1}$. The individual systems themselves are complex, consisting of several discrete cyber and physical components, which must be operational and connected to the network. The individual components of $S_{i}$ may be disabled or disconnected, and $S_{i}$ as a system may be disconnected, by cyber and physical attacks on the components. We formulate the problem of ensuring the infrastructure performance as a game between an attacker that launches cyber or physical attacks on the components and a provider that reinforces them to withstand the attacks. Since both attacks and reinforcements incur costs, the two players both weight the costs with expected benefits by minimizing utility functions. We derive Nash Equilibrium (NE) conditions that provide expressions for individual system survival probabilities and also the expected capacity specified by the total number of operational components. This paper extends and presents a unified view of the partial results presented in earlier conference papers on sum- and product-form utilities [1], composite utilities [2,3] and multi-site cloud infrastructures [4].

The components can be reinforced to survive direct attacks, but they can still be unavailable due to attacks on other components. For example, a super computer at a site may be hardened against cyber attacks, but can still be made unavailable by cutting fiber connections to the site. On the other hand, we consider that non-reinforced cyber and physical components will be disabled by direct cyber and physical attacks, respectively. Furthermore, in addition to within system $S_{i}$, the effects of attacks on its components may propagate to components of other systems $S_{j}$, j≠i. Thus, both the correlations between components within individual systems and those between systems represent the propagation of disruptions across the infrastructure. The infrastructure provider is tasked with developing strategies to choose a number of components of each system to reinforce against the attacks by taking into account both types of correlations.

Let $n_{i}$ denote the number of components of $S_{i}$, i=1,2,…,N+1, of which $y_{i}$ and $x_{i}$ denote the number of components attacked and reinforced, respectively. Let $P_{i}$ be the survival probability of $S_{i}$ and $P_{I}$ be the survival probability of the entire infrastructure. Furthermore, let $S_{-i}$ denote the infrastructure without $S_{i}$ and $P_{-i}$ be its survival probability. The relative importance of $S_{i}$ is captured by the aggregate failure correlation function $C_{i}$ given by the failure probability of $S_{-i}$ given the failure of $S_{i}$. Under the asymmetric network conditions, the specific role of the network is specified by two conditions: (a) $C_{N+1}=1$ indicates that network failure will disrupt the entire infrastructure; and (b) $C_{i}=0$, for i=1,2,…,N, indicates that disruptions of individual systems are uncorrelated. The correlations between components of individual systems are captured by simple first-order differential conditions on $P_{i}$ using the system multiplier functions that capture correlations within systems and also abstract the effects of system-level parameters [5]. This two-level characterization helps to conceptualize the basic correlations in infrastructures, such as cloud computing and smart grid infrastructures and provides insights into the needed defense strategies by naturally “separating” the system-level and component-level aspects.

A game between an attacker and a provider involves balancing the costs of attacks and reinforcements of systems, given by $L_{A}\left(y_{1},\ldots ,y_{N+1}\right)$ and $L_{D}\left(x_{1},\ldots ,x_{N+1}\right)$, respectively, with the survival probability of the infrastructure. We consider that the provider minimizes the composite utility function given by:

$$\begin{array}{cc} U_{D}\left(x_{1},\ldots ,x_{N+1},y_{1},\ldots ,y_{N+1}\right)= & F_{D,G}\left(x_{1},\ldots ,x_{N+1},y_{1},\ldots ,y_{N+1}\right)G_{D}\left(x_{1},\ldots ,x_{N+1},y_{1},\ldots ,y_{N+1}\right)\\ & +F_{D,L}\left(x_{1},\ldots ,x_{N+1},y_{1},\ldots ,y_{N+1}\right)L_{D}\left(x_{1},\ldots ,x_{N+1}\right) \end{array}$$

where the first product term corresponds to the reward and the second product term corresponds to the cost. Within the product terms, $F_{D,G}$ and $F_{D,L}$ are the reward and cost multipliers, respectively, of the provider, and $G_{D}$ and $L_{D}$ represent the reward and cost terms, respectively, of keeping the infrastructure operational. Similarly, we consider that the attacker minimizes:

$$\begin{array}{cc} U_{A}\left(x_{1},\ldots ,x_{N+1},y_{1},\ldots ,y_{N+1}\right)= & F_{A,G}\left(x_{1},\ldots ,x_{N+1},y_{1},\ldots ,y_{N+1}\right)G_{A}\left(x_{1},\ldots ,x_{N+1},y_{1},\ldots ,y_{N+1}\right)\\ & +F_{A,L}\left(x_{1},\ldots ,x_{N+1},y_{1},\ldots ,y_{N+1}\right)L_{A}\left(y_{1},\ldots ,y_{N+1}\right) \end{array}$$

where $F_{A,G}$ and $F_{A,L}$ are the reward and cost multipliers, respectively, of the attacker, and $G_{A}$ and $L_{A}$ represent the reward and cost terms of disrupting the infrastructure operation, respectively. The expected capacity of the infrastructure is the expected number of available components, given by:

$$N_{I}=\sum_{i=1}^{N}n_{i}P_{i}$$

which reflects the part of the infrastructure that survives the attacks. In the example of the cloud infrastructure, it represents the number of operational servers that are available to users on average.

The composite utility function can be specialized to obtain sum-form and product-form utilities by using appropriate terms, as summarized in Table 1, and their choice represents different values in keeping the infrastructure operational:

(a) The sum-form utility function is given by:
  $$\begin{array}{c} U_{D+}=-\left[P_{I}\left(x_{1},\ldots ,x_{N+1},y_{1},\ldots ,y_{N+1}\right)\right]g_{D}+L_{D}\left(x_{1},\ldots ,x_{N+1}\right) \end{array}$$
  which will be minimized by the provider. The scalar $g_{D}\geq 0$ represents the benefit of keeping the infrastructure operational such as income from an operational cloud computing infrastructure. Thus, the sum-form represents a weak coupling between gain and cost terms, since the effect of their minimization on the utility function is independent. For a provider, this form is used when explicit “gain” in keeping the infrastructure up can be identified and balanced against the cost.
(b) The product-form utility function is given by:
  $$\begin{array}{c} U_{D\times }=\left[1-P_{I}\left(x_{1},\ldots ,x_{N+1},y_{1},\ldots ,y_{N+1}\right)\right]\times L_{D}\left(x_{1},\ldots ,x_{N+1}\right) \end{array}$$
  which will be minimized by the provider; it represents the “wasted” cost to the provider since it is the expected cost under the condition that the infrastructure fails. Thus, the product-form represents a strong coupling between probability and cost terms, since the effect of minimization of one term gets multiplied by the other. This utility is used when the main goal of the provider is to keep the infrastructure up with the cost incurred, since there is no explicit ”gain” term.

The Nash Equilibrium (NE) conditions based on the utility functions can used to estimate $x_{i}$’s for the provider using various methods [6,7]. Our objective in this paper is to show that critical insights can be gained by deriving estimates of system survival probabilities and expected capacity explicitly in terms of various correlations, without relying on explicit solutions for $x_{i}$’s. The differences in the goals of sum- and product-form utilities lead to qualitatively different defense strategies, which are derived separately in earlier works, and the corresponding expressions for the survival probabilities that are structurally different [5,8]. We show that under the asymmetric network conditions, NE conditions of this game lead to expressions for $P_{i}$’s and $N_{I}$ with the same structure. In particular, the estimates of $P_{i}$ for sum-form and product-form utilities have the same expression in Theorem 3 except for one term, given by $\xi_{i}^{+}=\frac{1}{g_{D}}\frac{\partial L_{D}}{\partial x_{i}}$ and $\xi_{i}^{\times }=\left(1-P_{I}\right)\frac{\partial \ln L_{D}}{\partial x_{i}}=\frac{\left(1-P_{I}\right)}{L_{D}}\frac{\partial L_{D}}{\partial x_{i}}$. To consider the case where the sum-form and the product-form utilities are equivalent, we equate the two terms and obtain the following “equivalent” gain term of the sum-form:

$$g_{D}=\frac{L_{D}}{\left(1-P_{I}\right)}=L_{D}\left[1+\sum_{i=1}^{\infty }P_{I}^{i}\right]$$

for $0<P_{I}<1$, which is an increasing function in both $P_{I}$ and $L_{D}$; or, equivalently, we have, $P_{I}=1-L_{D}/g_{D}$. This similarity is striking since the sum-form and product-form utilities represent two quite different objectives.

The composite utility functions lead to simple expressions for $P_{i}$, i=1,2,…,N, and $N_{I}$ at NE, which subsume the above cases. In general, the dependence of $P_{i}$ on cost terms and aggregate correlation functions, as well as their partial derivatives, is presented in a compact form by using the composite gain-cost and composite multiplier terms (defined in Section 4). The expected capacity at NE is expressed in terms of cost term $L_{D}$ and its derivative, the aggregate correlation functions $C_{i}$, i=1,2…,N+1, and the system multiplier functions, $\Lambda_{i}$, i=1,2…,N+1 (defined in Section 3.2). The expression provides critical information on the dependence of the expected capacity on system parameters, in particular $C_{i}$ and $\Lambda_{i}$, and utility functions. Furthermore, by decomposing the system models into sub-models, such as cyber and physical sub-models, finer relationships can be inferred between system parameters, such as refined versions of $C_{i}$ and $\Lambda_{i}$, and the expected capacity. We apply these results to a simplified model of cloud computing infrastructure with multiple server sites connected over a communications network.

The organization of this paper is as follows. We describe related work in Section 2. In Section 3, we describe the infrastructure model along with the aggregate correlation function and differential conditions on system survival probabilities. We present our game-theoretic formulation using sum-form, product-form and composite utility functions in Section 4 and derive NE conditions and estimates for the system survival probabilities and expected capacity. We apply the analytical results to a model of cloud computing infrastructure in Section 5. We present conclusions in Section 6.

## 2. Related Work

Critical infrastructures of power grids, cloud computing and transportation systems provide vital public and private services [9,10]. They depend on complex communications networks that connect the constituent systems, which by themselves consist of many disparate cyber and physical components [10]. The communications network plays a very critical role in these infrastructures [11], in some ways more so than the constituent systems, and its failure can significantly degrade the entire infrastructure [12,13]. These infrastructures are under increasing cyber and physical attacks, which the providers are required to counter by applying defense measures and strategies.

By capturing the interactions between providers and attackers of these infrastructures, game-theoretic methods have been extensively applied to develop the needed defense strategies [14,15,16], which attempt to ensure continued infrastructure operations in the presence of evolving threats [17]. Partial differential equations and discrete component models have been used in several of these infrastructures to model the physical and cyber systems [18] in formulating the underlying games. The game-theoretic formulations and the solutions developed for such infrastructures are quite varied and extensive. They include: multiple-period games that address multiple time-scales of system dynamics [19]; incomplete information games that account for partial knowledge about the system dynamics and attack models [20]; and multiple-target games that account for possibly competing objectives [21].

A comprehensive review of the defense and attack models in various game-theoretic formulations has been presented in [22]. Recent interest in cyber and cyber-physical systems led to the application of game theory to a variety of cyber security scenarios [16,23] and, in particular, for securing cyber-physical networks [24] with applications to power grids [11,25,26,27].

The system availability, reliability and robustness aspects can be explicitly integrated into the game formulations [14] for infrastructures such as power grids, cloud computing and transportation systems. In particular, discrete models of cyber-physical infrastructures have been studied in various forms under Stackelberg game formulations [28]. A subclass of these models using the number of cyber and physical components that are attacked and reinforced as the main variables have been studied in [29]. These models characterize infrastructures with a large number of components and are coarser compared to the models that consider the attacks and reinforcements of individual cyber and physical components. Various forms of correlation functions [5,8,29] are used in these works to capture the dependencies between the survival probabilities of constituent systems, such as the cyber and physical sub-infrastructures.

Complex interacting collections of systems have been studied using game-theoretic formulations in [30], and their two-level correlations have been studied using the sum-form utility functions in [5] and the product-form utility functions in [8]. The sum-form utility represents a gain-centric priority, wherein an independent gain term weighted by $P_{I}$ represents the gain to be maximized by the provider. The product-form utility, on the other hand, represents a cost-centric priority, wherein the expected cost is to be minimized. The sum-form utility function [5] and the product-form utility function [8] are considered separately for a generic version of this game, wherein all systems play a similar role, unlike the asymmetric role of the network considered here. In terms of analysis, these two formulations have a certain degree of commonality, but there are also differences; in particular, estimates of $P_{I}$ can be obtained somewhat directly for the product-form as shown in [8]. These two utility functions also lead to qualitatively different defense strategies, and in particular, $P_{I}$ appears explicitly in the sensitivity estimates of system survival probabilities in product-form, but not in sum-form. These two utility functions are unified in [2], and the sum-form utility function has been studied under the asymmetric role of the communications network in [1].

The infrastructures for smart energy grids, cloud computing and intelligent transportation systems are composed of complex constituent systems that rely on communications networks. For wide-area operations, these networks play a critical asymmetric role of providing the vital connectivity needed for continued infrastructure operations. The asymmetric network correlations have been incorporated into multiple system infrastructures for sum-form and product-form utilities in [1], and these two works are unified in [3] by using the composite utility functions. The multi-site cloud computing infrastructure was discussed as an example for sum-form and product-form utility functions in [1] and composite utility functions in [3], wherein the network plays a critical asymmetric role. This model is further extended by including an HVAC system in [4], and also, additional details of NE conditions and capacity estimates are provided. In this paper, we consolidate these results and present a unified treatment of the sum-form, product-form and composite utilities under asymmetric network correlation conditions. For multi-site cloud infrastructures, we explicitly relate these utility functions and interpret the abstract definitions of correlation functions and system multiplier functions in terms of systems and their components.

## 3. Discrete System Models

We consider infrastructures with constituent systems consisting of discrete components [5,8] and connected over a communications network [1]. We first consider the correlations at the systems and network levels and then consider the correlations between the components of individual systems.

### 3.1. System-Level Correlations

The correlations between systems, including the network, in these infrastructures are characterized in terms of their survival probabilities as follows.

**Condition** **1.** *Aggregate correlation function: Let $C_{i}$ denote the failure probability of the rest of the infrastructure $S_{-i}$ given the failure of $S_{i}$, and let $C_{-i}$ denote the failure probability of $S_{i}$ given the failure of $S_{-i}$ such that:*

$$C_{i}\left(1-P_{i}\right)=C_{-i}\left(1-P_{-i}\right)$$

*for i=1,…,N+1. Then, the survival probability of the infrastructure is given by:*

$$\begin{array}{c} P_{I}=P_{i}+P_{-i}-1+C_{i}\left(1-P_{i}\right)=P_{i}+P_{-i}-1+C_{-i}\left(1-P_{-i}\right)\;□ \end{array}$$

The aggregate failure correlation function captures the interdependence of the rest of the system $S_{-i}$ on the failure of $S_{i}$, which can be illustrated using the following special cases.

(a) Asymmetric network: In a cloud computing infrastructure, consider that the fiber connections to *N* sites, each with *l* servers, constitute the network system $S_{F}=S_{N+1}$. Then, we have:
  $$P_{-F}=1-l\left(1-P_{F}\right)/K$$
  where *K* is a normalization constant, since the fiber failure rate is amplified by *l* in rendering the servers unavailable. Thus, we have:
  $$P_{I}=\left[1-\left(C_{F}-l/K\right)\right]P_{F}+C_{F}-l/K$$
(b) Statistical independence: The system failures satisfy a statistical independent condition given by $C_{i}=1-P_{-i}$, indicating that the failure probability of $S_{-i}$ is not dependent on $P_{i}$. This condition in turn leads to $P_{I}=P_{i}P_{-i}$, which indicates the statistical independence of the survival processes of $S_{i}$ and $S_{-i}$. More generally, if $C_{i}>1-P_{-i}$, the failures in $S_{-i}$ are positively correlated with failures in $S_{i}$, that is they occur with a higher probability following the latter. If we denote the failure probability of $S_{i}$ by $P_{\bar{i}}$, then we have $P_{\bar{-i}|\bar{i}}>P_{\bar{-i}}$, or equivalently, failure in $S_{i}$ leads to a higher probability of failure in $S_{-i}$. If $C_{i}<1-P_{-i}$, failures in $S_{-i}$ are negatively correlated with the latter failures, that is $P_{\bar{-i}|\bar{i}}<P_{\bar{-i}}$.
(c) Definite failure: In another case, when the failure of $S_{i}$ leads to a definite failure of the rest of the infrastructure, we have $C_{i}\left(P_{i}\right)=1$ such that $P_{I}=P_{-i}$. This condition indicates that the infrastructure survival probability solely depends on the marginal failure probability of $S_{-i}$.
(d) ORsystems: The OR systems as modeled in [29] correspond to the special case N=2 where the infrastructure consists of uncorrelated cyber and physical systems (denoted by i=C and −i=P, respectively) that can be independently analyzed. For OR systems, the failure probabilities of $S_{i}$ and $S_{-i}$ are uncorrelated such that $C_{i}=C_{-i}=0$, and hence, we have $P_{\bar{i}\cup \bar{-i}}=P_{\bar{i}}+P_{\bar{-i}}$ or equivalently $P_{\bar{i}\cap \bar{-i}}=0$. Thus, we have $P_{I}=P_{i}+P_{-i}-1$. We apply this condition next in Condition 2 for *N* systems considered in this paper.

The important asymmetric role of the communications network is characterized using the following condition.

**Condition** **2.** *Asymmetric network and uncorrelated systems conditions: The aggregated correlation functions of $S_{i}$, i=1,2,…,N+1, satisfy the conditions: (i) for the network $S_{N+1}$, we have $C_{N+1}=1$, and (ii) for the constituent systems, we have $C_{i}=0$, i=1,2,…,N.* ☐

Part (i) of Condition 2 leads to $P_{I}=P_{-(N+1)}$, which indicates the role of the rest of infrastructure $S_{-(N+1)}$ without the network; namely, its survival probability is the same as that for server sites together. Part (ii) of Condition 2 leads to $P_{I}=P_{i}+P_{-i}-1$, i=1,2,…,N, which linearly depends on each of the failure probabilities of the constituent system $S_{i}$ and the rest of infrastructure $S_{-i}$. It is important to note that although there are direct correlations between the site failures zero (Part (ii) above), these site failures are still indirectly related through the network. In particular, the failures of $S_{i}$ and $S_{j}$, which are parts of $S_{-(N+1)}$, are correlated with the network via $C_{N+1}$; for example, they both become simultaneously unavailable when the wide-area network fails.

The effects of reinforcements and attacks on host sites and wide-area networks can be separated using the following two conditions:

(i) the first condition, $\frac{\partial P_{-i}}{\partial x_{i}}=0$ for i=1,2,…,N, indicates that reinforcing the server site $S_{i}$ does not directly impact the survival probability of other sites or networks; and
(ii) the second condition, $\frac{\partial P_{i}}{\partial x_{j}}=0$ for i=1,2,…,N+1, j=1,2,…,N and j≠i, indicates that reinforcing server sites or network $S_{j}$ does not directly impact the survival probability of server sites or network $S_{i}$.

While the reinforcements to individual server sites or networks are not directly reflected in other systems, their failures may still be correlated due to the underlying system structures as reflected in the aggregated correlation function of the network $C_{N+1}$. These system-level considerations for the provider are captured by the following condition, which is obtained by differentiating $P_{I}$ in Condition 1 with respect to $x_{i}$ and ignoring the terms corresponding to Parts (i) and (ii) above.

**Condition** **3.** *De-coupled reinforcement effects: For $P_{I}$ in Condition 1, we have for i=1,2,…,N+1,*

$$\frac{\partial P_{I}}{\partial x_{i}}=\left(1-C_{i}\right)\frac{\partial P_{i}}{\partial x_{i}}+\left(1-P_{i}\right)\frac{\partial C_{i}}{\partial x_{i}}$$

*for the provider.* ☐

The condition captures the effect on the increment in $P_{I}$ as a result of the change in the number of reinforced components $x_{i}$ of $S_{i}$. It is the sum of (i) the increment in individual system survival probability $P_{i}$ weighted by “non-correlation” term $\left(1-C_{i}\right)$ and (ii) the increment in correlation $C_{i}$ weighted by the failure probability $1-P_{i}$ of $S_{i}$. For the sites $S_{i}$, i=1,2,…,N, we have:

$$\frac{\partial P_{I}}{\partial x_{i}}=\frac{\partial P_{i}}{\partial x_{i}}+\left(1-P_{i}\right)\frac{\partial C_{i}}{\partial x_{i}}$$

For the network $S_{N+1}$, we have:

$$\frac{\partial P_{I}}{\partial x_{N+1}}=\left(1-P_{N+1}\right)\frac{\partial C_{N+1}}{\partial x_{N+1}}$$

Under Condition 2, $C_{i}$ is constant, but its partial derivatives with respect to $x_{i}$ could be non-zero, as other parameters change to keep it constant.

### 3.2. Component-Level Correlations

The system survival probabilities satisfy the following differential condition that specifies the correlations at the component level [5,31].

**Condition** **4.** *System multiplier functions: The survival probabilities $P_{i}$ and $P_{-i}$ of system $S_{i}$ and $S_{-i}$, respectively, satisfy the following conditions: there exist system multiplier functions $\Lambda_{i}$ and $\Lambda_{-i}$ such that:*

$$\begin{array}{c} \frac{\partial P_{i}}{\partial x_{i}}=\Lambda_{i}\left(x_{1},\ldots ,x_{N+1},y_{1},\ldots ,y_{N+1}\right)P_{i}\;\;\;\;\;\;\;\;\mathrm{and}\;\;\;\;\;\;\;\frac{\partial P_{-i}}{\partial x_{i}}=\Lambda_{-i}\left(x_{1},\ldots ,x_{N+1},y_{1},\ldots ,y_{N+1}\right)P_{-i} \end{array}$$

*for i=1,2,…,N+1.* ☐

The derivative in the above condition is linear in $P_{i}$ for $\Lambda_{i}=1$ and is faster than linear if $\Lambda_{i}>1$ and slower than linear if $\Lambda_{i}<1$. These system multiplier functions capture the underlying system structure including its parameters, in addition to the game variables $x_{i}$’s and $y_{i}$’s. For example, in the case of multi-site server infrastructure, $\Lambda_{i}$ in Section 5.2 depends on the number of severs $l_{i}$ at site *i*. This somewhat abstract condition enables us to capture such a structure in a generic manner and indeed is satisfied in two special cases studied extensively in the literature.

(a) Statistically independent components: The special case when component survival probabilities are statistically independent with and without reinforcements has been studied in [31]. Let $p_{i|R}$ and $p_{i|W}$ denote the conditional survival probability of a component of $S_{i}$ with and without reinforcement, respectively. Under the statistical independence condition of component failures, the probability that $S_{i}$ with $n_{i}$ components survives the attacks is:
  $$P_{i}=p_{i|R}^{x_{i}}p_{i|W}^{n_{i}-x_{i}}$$
  as in [31], or equivalently:
  $$\ln P_{i}=n_{i}\ln p_{i|W}+x_{i}\ln\left(\frac{p_{i|R}}{p_{i|W}}\right)$$
  By differentiating the equation with respect to $x_{i}$, we obtain:
  $$\frac{\partial P_{i}}{\partial x_{i}}=\ln\left(\frac{p_{i|R}}{p_{i|W}}\right)P_{i}$$
  The condition for the faster than linear derivative is $\ln\left(\frac{p_{i|R}}{p_{i|W}}\right)>1$ or equivalently $p_{i|R}>ep_{i|W}$, where *e* is the base of the natural logarithm. The condition that the survival probability of a reinforced component is higher than that of a non-reinforced component, but less than $ep_{i|W}$, namely, $ep_{i|W}>p_{i|R}>p_{i|W}$, corresponds to only the slower than linear derivative.
(b) Contest survival functions: The contest survival functions are to express $P_{i}$ in [30] such that $P_{i}=\frac{\xi +x_{i}}{\xi +x_{i}+y_{i}}$ for a suitably-selected slack variable ξ, which in turn leads to:
  $$\frac{\partial P_{i}}{\partial x_{i}}=\left[\frac{y_{i}}{\left(\xi +x_{i}+y_{i}\right)\left(\xi +x_{i}\right)}\right]P_{i}$$
  The condition for the slower than linear derivative is:
  $$y_{i}\left[1-\left(x_{i}+\xi \right)\right]<{\left(\xi +x_{i}\right)}^{2}$$
  which is satisfied for larger values of $x_{i}$ sufficient to make the left-hand side negative.

## 4. Game Theoretic Formulation

The provider’s objective is to make the infrastructure resilient by reinforcing $x_{i}$ components of $S_{i}$ to optimize the utility function. Similarly, the attacker’s objective is to disrupt the infrastructure by attacking $y_{i}$ components of $S_{i}$ to optimize the corresponding utility function. NE conditions are derived by equating the corresponding derivatives of the utility functions to zero, which yields:

$$\frac{\partial U_{D}}{\partial x_{i}}=\left(G_{D}\frac{\partial F_{D,G}}{\partial P_{I}}+L_{D}\frac{\partial F_{D,L}}{\partial P_{I}}\right)\frac{\partial P_{I}}{\partial x_{i}}+F_{D,G}\frac{\partial G_{D}}{\partial x_{i}}+F_{D,L}\frac{\partial L_{D}}{\partial x_{i}}=0$$

for i=1,2,…,N+1 for the provider. We define:

$$L_{G,L}^{D}=G_{D}\frac{\partial F_{D,G}}{\partial P_{I}}+L_{D}\frac{\partial F_{D,L}}{\partial P_{I}}$$

as the composite gain-cost term, wherein the gain $G_{D}$ and cost $L_{D}$ are “amplified” by the derivatives of their corresponding multiplier functions with respect to $P_{I}$. We then define:

$$F_{G,L}^{D,i}=F_{D,G}\frac{\partial G_{D}}{\partial x_{i}}+F_{D,L}\frac{\partial L_{D}}{\partial x_{i}}$$

as the composite multiplier term, wherein the gain multiplier $F_{D,G}$ and cost multiplier $F_{D,L}$ are “amplified” by the derivatives of their corresponding gain and cost terms with respect to $x_{i}$, i=1,2,…,N+1, respectively. These two terms lead to the compact NE condition $\frac{\partial P_{I}}{\partial x_{i}}=-\frac{F_{G,L}^{D,i}}{L_{G,L}^{D}}.$ These NE conditions can be used to solve for $x_{i}$’s using available methods whose complexity depends on the details of gain and cost terms [14,15,16]. Indeed, different methods and trade-offs may be required to derive such solutions by exploiting the details of infrastructure [7]. We show in the next section that estimates for system survival probabilities and expected capacity can be obtained without explicitly solving for $x_{i}$’s, and yet, they provide valuable qualitative insights about the infrastructure. Various terms of the composite utility function specialized to sum-form and product-form utilities are shown in Table 2, which are considered separately in Section 4.3.

### 4.1. OR Systems

The OR systems [31] constitute a sub-class of abstract infrastructures where simultaneous failures of two or more systems are extremely unlikely, namely their probability is zero. These abstract models are used to illustrate the simplifications that result from ignoring the correlations and are generally used for analysis purposes. Here, OR systems ignore the asymmetric role played by the communications network. These systems are simpler to analyze due to the absence of system-level correlation terms, and they serve as base study cases when the correlations can be ignored. Indeed, an estimate of $P_{i}$ can be derived as a simple ratio of the gain-cost gradient and system multiplier function $\Lambda_{i}$. Using $P_{S}=P_{i}+P_{-i}-1$, we obtain:

$$\frac{\partial P_{i}}{\partial x_{i}}=-\frac{F_{G,L}^{D,i}}{L_{G,L}^{D}}=-\Theta_{i}\left(x_{1},\ldots ,x_{N},y_{1},\ldots ,y_{N}\right)$$

where $\Theta_{i}\left(\cdot \right)$ is called the scaled gain-cost gradients of system $S_{i}$. Then, Condition 4 provides us an estimate for the survival probability of $S_{i}$ as the ratio of the scaled gain-cost gradient and the system multiplier function given by:

$${\tilde{P}}_{i;D}\left(x_{1},\ldots ,x_{N},y_{1},\ldots ,y_{N}\right)=-\frac{\Theta_{i}\left(x_{1},\ldots ,x_{N},y_{1},\ldots ,y_{N}\right)}{\Lambda_{i}\left(x_{1},\ldots ,x_{N},y_{1},\ldots ,y_{N}\right)}$$

for i=1,2,…,N. These estimates for individual systems depend mainly on the corresponding scaled gain-cost gradients and thus represent a “separation” of the individual systems at this level. In this sense, OR systems constitute an important analytical case wherein the correlations between the individual systems may be ignored. In addition, these estimates provide the sensitivity information of the survival probabilities of the individual systems with respect to various quantities of $S_{i}$. In particular, the survival probability estimate ${\tilde{P}}_{i;D}$ is proportional to the corresponding weighted cost and reward functions and inversely proportional to their weighted derivatives. This seemingly counter-intuitive trend applies only to the set of Nash equilibria and not to the overall system behavior. In the rest of the paper, we denote $\Lambda_{i}\left(x_{1},\ldots ,x_{N},y_{i},\ldots ,y_{N}\right)$ and $\Theta_{i}\left(x_{1},\ldots ,x_{N},y_{i},\ldots ,y_{N}\right)$, by $\Lambda_{i}$ and $\Theta_{i}$, respectively, to simplify the notation.

### 4.2. System Survival Probabilities and Expected Capacity

We now derive estimates for $P_{i}$ at NE using aggregated correlation functions and their partial derivatives to infer qualitative information about their sensitivities to different parameters.

**Theorem** **1.** *Survival probability estimates: Under Conditions 1, 3 and 4, estimates of the survival probability of system $S_{i}$, for i=1,2,…,N+1, are given by:*

$${\hat{P}}_{i;D}=\frac{\frac{\partial C_{i}}{\partial x_{i}}+\frac{F_{G,L}^{D,i}}{L_{G,L}^{D}}}{\frac{\partial C_{i}}{\partial x_{i}}-\left(1-C_{i}\right)\Lambda_{i}}$$

*for i=1,2,…,N+1 under the condition: $C_{i}<1$ or $\frac{\partial C_{i}}{\partial x_{i}}\neq 0$. Under the asymmetric network correlation coefficient $C_{N+1}=1$, the survival probability of the network is given by:*

$$P_{-(N+1);D}=-\frac{1}{\Lambda_{-(N+1)}}\frac{F_{G,L}^{D,N+1}}{L_{G,L}^{D}}$$

**Proof.**  Our proof is based on deriving NE conditions for the utility function. At NE, we have:

$$\frac{\partial P_{I}}{\partial x_{i}}=-\frac{F_{G,L}^{D,i}}{L_{G,L}^{D}}$$

Then, using the equation in Condition 3 and $\frac{\partial P_{i}}{\partial x_{i}}=\Lambda_{i}P_{i}$ from Condition 4, we have:

(1) $$\left(1-C_{i}\right)\Lambda_{i}P_{i;D}+\left(1-P_{i;D}\right)\frac{\partial C_{i}}{\partial x_{i}}=-\frac{F_{G,L}^{D,i}}{L_{G,L}^{D}}$$

Under the condition $C_{i}<1$ or $\frac{\partial C_{i}}{\partial x_{i}}\neq 0$, we have $\frac{\partial C_{i}}{\partial x_{i}}-\left(1-C_{i}\right)\Lambda_{i}\neq 0$, and hence, we obtain:

$$P_{i;D}=\frac{\frac{\partial C_{i}}{\partial x_{i}}+\frac{F_{G,L}^{D,i}}{L_{G,L}^{D}}}{\frac{\partial C_{i}}{\partial x_{i}}-\left(1-C_{i}\right)\Lambda_{i}}$$

for i=1,2,…,N+1.

Consider the survival probability of the infrastructure; under the asymmetric network condition, we have $C_{N+1}=1$ and $\frac{\partial C_{N+1}}{\partial x_{N+1}}=0$, which imply that the condition $C_{i}<1$ or $\frac{\partial C_{i}}{\partial x_{i}}\neq 0$ is not satisfied; hence, the above formula cannot be used directly since the denominator $\frac{\partial C_{i}}{\partial x_{i}}-\left(1-C_{i}\right)\Lambda_{i}=0$. Instead, using $C_{N+1}=1$ in Condition 1, we obtain $P_{I}=P_{-(N+1)}$, which implies:

$$\frac{\partial P_{I}}{\partial x_{N+1}}=\frac{\partial P_{-(N+1)}}{\partial x_{N+1}}$$

Then, the NE condition is given by:

$$\frac{\partial P_{I}}{\partial x_{N+1}}=\frac{\partial P_{-(N+1);D}}{\partial x_{N+1}}=\Lambda_{-(N+1)}P_{-(N+1);D}=-\frac{F_{G,L}^{D,N+1}}{L_{G,L}^{D}}$$

which completes the proof. ☐

The system survival probability estimates ${\hat{P}}_{i;D}$ provide qualitative information about the effects of various parameters including aggregated correlation coefficient $C_{i}$, system multiplier functions $\Lambda_{i}$, composite gain-cost $L_{G,L}^{D}$ and composite multiplier $F_{G,L}^{D,i}$; note that the estimates may not necessarily lie within the range [0,1]. In particular, ${\hat{P}}_{i;D}$ (i) increases and decreases with $F_{G,L}^{D,i}$ and $L_{G,L}^{D}$, respectively, (ii) increases with $\Lambda_{i}$ and (iii) depends both on $C_{i}$ and its derivative for i=1,2,…,N. For the network, $P_{-(N+1);D}$ is in a simpler form since $C_{N+1}=1$.

We now consider that the asymmetric role played by the network described in Condition 2, namely its failure, renders entire infrastructure unavailable; also, failures of individual systems are uncorrelated with others. The following theorem provides a single, simplified expression for the expected capacity under these conditions.

**Theorem** **2.** *Expected capacity under asymmetric network correlations: Under Conditions 1–4, the expected capacity is given by:*

$$N_{I}=\sum_{i=1}^{N}\left(-\frac{n_{i}}{\Lambda_{i}}\frac{F_{G,L}^{D,i}}{L_{G,L}^{D}}\right)$$

**Proof.**  Under Part (ii) of Condition 2, Equation (1) in the proof of Theorem 1 simplifies to the equation:

$$\Lambda_{i}P_{i;D}=-\frac{F_{G,L}^{D,i}}{L_{G,L}^{D}}$$

for i=1,2,…,N. Thus, we have $P_{i}=-\frac{1}{\Lambda_{i}}\frac{F_{G,L}^{D,i}}{L_{G,L}^{D}}$, which provides the expression for $N_{I}$. ☐

This condition indicates that lower $L_{G,L}^{D}$ and higher composite multiplier $F_{G,L}^{D,i}$ lead to lower expected capacity. Typically, the composite gain-cost $L_{G,L}^{D}$ is negative (e.g., $-g_{D}$ for sum-form) since it is minimized by the provider; thus, its lower value is more negative and has a higher magnitude. Furthermore, larger values of $\Lambda_{i}$ also lead to lower expected capacity. In particular, the condition $\Lambda_{i}>1$, called the faster than linear growth of $\frac{\partial P_{i}}{\partial x_{i}}$, leads to lower expected capacity. This seems counter-intuitive since faster improvement in $P_{i}$ due to the increase in $x_{i}$ leads to lower expected capacity, but note that it only characterizes the states that satisfy NE conditions.

### 4.3. Sum-Form and Product-Form Utility Functions

The NE conditions for sum-form and product-form utilities are derived by equating the corresponding derivatives to zero, which yields the following conditions, respectively:

$$\frac{\partial U_{D+}}{\partial x_{i}}=\frac{\partial P_{I}}{\partial x_{i}}g_{D}-\frac{\partial L_{D}}{\partial x_{i}}=0\;\;\;\;\;\;\;\;\mathrm{and}\;\;\;\;\;\;\;\;\frac{\partial U_{D\times }}{\partial x_{i}}=-\frac{\partial P_{I}}{\partial x_{i}}L_{D}+\left(1-P_{I}\right)\frac{\partial L_{D}}{\partial x_{i}}=0$$

for i=1,2,…,N+1 for the provider.

We now derive estimates for $P_{i}$ at NE using partial derivatives of the cost and failure correlation functions to infer qualitative information about their sensitivities to different parameters.

**Theorem** **3.** *Under Conditions 1, 3 and 4, estimates of the survival probability of system $S_{i}$, for i=1,2,…,N+1, are given by:*

$${\hat{P}}_{i;D}^{A}=\frac{\frac{\partial C_{i}}{\partial x_{i}}-\xi_{i}^{A}}{\frac{\partial C_{i}}{\partial x_{i}}-\left(1-C_{i}\right)\Lambda_{i}}$$

*where A=+ and A=× correspond to sum-form and product-form, respectively, such that:*

$$\xi_{i}^{A}=\left\{\begin{array}{cc} \frac{1}{g_{D}}\frac{\partial L_{D}}{\partial x_{i}} & if\;\;A=+\\ \left(1-P_{I}\right)\frac{\partial \ln L_{D}}{\partial x_{i}}, & if\;\;A=\times \end{array}\right.$$

*for i=1,2,…,N+1 under the condition: $C_{i}<1$ or $\frac{\partial C_{i}}{\partial x_{i}}\neq 0$. Under the asymmetric network correlation coefficient $C_{N+1}=1$, the survival probability of the network is given by:*

$$P_{-(N+1);D}^{A}=\frac{\xi_{N+1}^{A}}{\Lambda_{-(N+1)}}$$

*for A=+,×.*

**Proof.**  Our proof is based on deriving NE conditions separately for sum-form and product-form utility functions and then comparing them to identify their common structure and the difference terms. At NE, for the sum-form, we have:

$$\frac{\partial P_{I}}{\partial x_{i}}=\frac{1}{g_{D}}\frac{\partial L_{D}}{\partial x_{i}}=\xi_{i}^{+}$$

Then, using the equation in Condition 3 and $\frac{\partial P_{i}}{\partial x_{i}}=\Lambda_{i}P_{i}$ from Condition 4, we have:

(2) $$\left(1-C_{i}\right)\Lambda_{i}P_{i;D}^{+}+\left(1-P_{i;D}^{+}\right)\frac{\partial C_{i}}{\partial x_{i}}=\frac{1}{g_{D}}\frac{\partial L_{D}}{\partial x_{i}}$$

Under the condition $C_{i}<1$ or $\frac{\partial C_{i}}{\partial x_{i}}\neq 0$, we have $\frac{\partial C_{i}}{\partial x_{i}}-\left(1-C_{i}\right)\Lambda_{i}\neq 0$, and hence, we obtain:

$$P_{i;D}^{+}=\frac{\frac{\partial C_{i}}{\partial x_{i}}-\frac{1}{g_{D}}\frac{\partial L_{D}}{\partial x_{i}}}{\frac{\partial C_{i}}{\partial x_{i}}-\left(1-C_{i}\right)\Lambda_{i}}=\frac{\frac{\partial C_{i}}{\partial x_{i}}-\xi_{i}^{+}}{\frac{\partial C_{i}}{\partial x_{i}}-\left(1-C_{i}\right)\Lambda_{i}}$$

for i=1,2,…,N+1. Similarly, for the product-form, we have:

(3) $$\frac{\partial P_{I}}{\partial x_{i}}=\left(1-P_{I}\right)\frac{1}{L_{D}}\frac{\partial L_{D}}{\partial x_{i}}=\left(1-P_{I}\right)\frac{\partial \ln L_{D}}{\partial x_{i}}=\xi_{i}^{\times }$$

Then, using the equation in Condition 3 and $\frac{\partial P_{i}}{\partial x_{i}}=\Lambda_{i}P_{i}$ from Condition 4, we have:

$$\left(1-C_{i}\right)\Lambda_{i}P_{i;D}^{\times }+\left(1-P_{i;D}^{\times }\right)\frac{\partial C_{i}}{\partial x_{i}}=\left(1-P_{I}\right)\frac{\partial \ln L_{D}}{\partial x_{i}}$$

Then, we have:

$$P_{i;D}^{\times }=\frac{\frac{\partial C_{i}}{\partial x_{i}}-\left(1-P_{I}\right)\frac{\partial \ln L_{D}}{\partial x_{i}}}{\frac{\partial C_{i}}{\partial x_{i}}-\left(1-C_{i}\right)\Lambda_{i}}$$

for i=1,2,…,N+1.

Consider the survival probability of the infrastructure; under the asymmetric network condition, we have $C_{N+1}=1$ and $\frac{\partial C_{N+1}}{\partial x_{N+1}}=0$, which imply that the condition $C_{i}<1$ or $\frac{\partial C_{i}}{\partial x_{i}}\neq 0$ is not satisfied; hence, the above formula cannot be used directly since the denominator $\frac{\partial C_{i}}{\partial x_{i}}-\left(1-C_{i}\right)\Lambda_{i}=0$. Instead, using $C_{N+1}=1$ in Condition 1, we obtain $P_{I}=P_{-(N+1)}$, which implies:

$$\frac{\partial P_{I}}{\partial x_{N+1}}=\frac{\partial P_{-(N+1)}}{\partial x_{N+1}}$$

Then, the NE condition for the sum-form is given by:

$$\frac{\partial P_{I}}{\partial x_{N+1}}=\frac{\partial P_{-(N+1);D}^{+}}{\partial x_{N+1}}=\Lambda_{-(N+1)}P_{-(N+1);D}^{+}=\frac{1}{g_{D}}\frac{\partial L_{D}}{\partial x_{N+1}}$$

Similarly, for the product-form, we obtain,

$$\frac{\partial P_{I}}{\partial x_{N+1}}=\Lambda_{-(N+1)}P_{-(N+1);D}^{\times }=\left(1-P_{I}\right)\frac{\partial \ln L_{D}}{\partial x_{N+1}}$$

which completes the proof. ☐

The estimates ${\hat{P}}_{i;D}$ above provide sensitivity information about the corresponding survival probabilities with respect to various parameters; note that the estimates may not necessarily lie within [0,1]. In particular, they qualitatively relate $P_{i}$ to the corresponding aggregate correlation function $C_{i}$ and its derivative, and also to $\Lambda_{i}$. These dependencies are identical for both sum-form and product-form utility functions. Indeed, the difference between the two formulae is captured by the single term $\xi_{i}^{A}$, which is proportional to the derivative term $\frac{\partial L_{D}}{\partial x_{i}}$ in both cases. The main difference is that $\xi_{i}^{\times }$ is an increasing function of $P_{I}$, whereas $\xi_{i}^{+}$ does not depend on $P_{I}$. Furthermore, the dependence on $L_{D}$ is different for these two terms. Since $\xi_{i}^{+}=\frac{1}{g_{D}}\frac{\partial L_{D}}{\partial x_{i}}$ and $\xi_{i}^{\times }=\left(1-P_{I}\right)\frac{1}{L_{D}}\frac{\partial L_{D}}{\partial x_{i}}$, the role of $g_{D}$ in the former is played by $L_{D}/\left(1-P_{I}\right)$ in the latter. Typically, $g_{D}$ is chosen as a constant in the sum-form, and $P_{I}$ is a function of $x_{i}$ and $y_{i}$.

We now consider that network failure renders the entire infrastructure unavailable, and the failure of individual systems is uncorrelated with others given by Condition 2. The following theorem provides a single, simplified expression for the expected capacity under these conditions.

**Theorem** **4.** *Asymmetric network correlations: Under Conditions 1–4, the expected capacity is given by:*

$$N_{I}^{A}=\sum_{i=1}^{N}\left(n_{i}\frac{\xi_{i}^{A}}{\Lambda_{i}}\right)$$

*where A=+ and A=× correspond to sum-form and product-form, respectively, such that:*

$$\xi_{i}^{A}=\left\{\begin{array}{cc} \frac{1}{g_{D}}\frac{\partial L_{D}}{\partial x_{i}} & if\;\;A=+\\ \left(1-P_{I}\right)\frac{\partial \ln L_{D}}{\partial x_{i}}, & if\;\;A=\times \end{array}\right.$$

*for i=1,2,…,N.*

**Proof.**  Under Part (ii) of Condition 2, Equations (2) and (3) in Theorem 3 simplify to the same equation $\Lambda_{i}P_{i;D}^{A}=\xi_{i}^{A}$ for A=+,× and i=1,2,…,N. Thus, we have $P_{i}^{A}=\frac{\xi_{i}^{A}}{\Lambda_{i}}$, which provides the expression for $N_{I}^{A}$. ☐

For the sum-form,

$$N_{I}^{+}=\sum_{i=1}^{N}\left(\frac{n_{i}\frac{\partial L_{D}}{\partial x_{i}}}{g_{D}\Lambda_{i}}\right)$$

indicates that higher gain $g_{D}$ leads to a lower number of operational components. For the product form,

$$N_{I}^{\times }=\left(1-P_{I}\right)\sum_{i=1}^{N}\left(\frac{n_{i}\frac{\partial L_{D}}{\partial x_{i}}}{L_{D}\Lambda_{i}}\right)$$

indicates that higher survival probability of the network leads to a lower number of operational components. The dependence on $\Lambda_{i}$ is similar in both cases, namely faster than linear leads to a lower number of available component, and vice versa. The dependence on $L_{D}$ is somewhat different due to its presence in the denominator for the product-form, even though $\frac{\partial L_{D}}{\partial x_{i}}$ appears in the numerator in both forms.

The expressions of $N_{I}$ for the composite utility are simpler due to the generality of the composite gain-cost and composite multiplier, which are complex by themselves in that the sum-form and product-form are subsumed by them as indicated in Table 1. Typically, the composite gain-cost $L_{G,L}^{D}$ is negative (e.g., $-g_{D}$ for the sum-form) since it is minimized by the provider; thus, its lower value is more negative and has a higher magnitude. Furthermore, larger values of $\Lambda_{i}$ also lead to lower expected capacity. In particular, the condition $\Lambda_{i}>1$, called the faster than linear growth of $\frac{\partial P_{i}}{\partial x_{i}}$, leads to lower expected capacity. This seems counter-intuitive since faster improvement in $P_{i}$ due to the increase in $x_{i}$ leads to lower expected capacity, but note that it only characterizes the states that satisfy NE conditions.

## 5. Multi-Site Server Infrastructure

**Figure 1:**
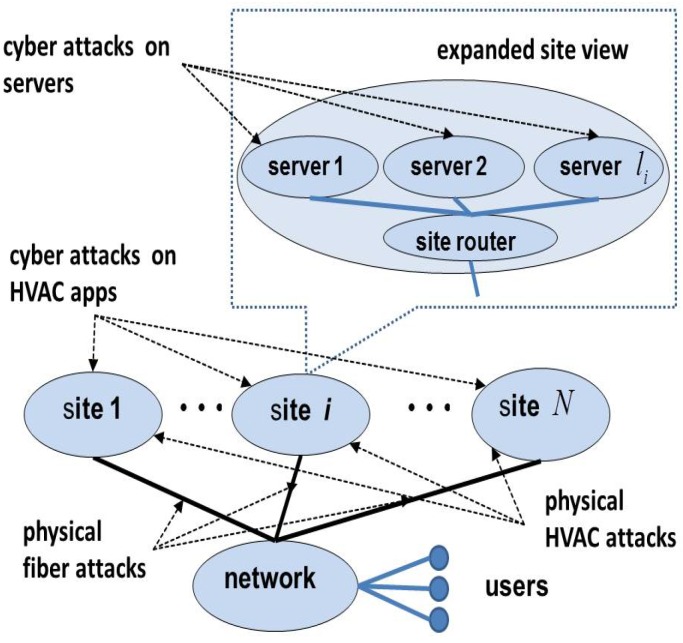
Cloud computing infrastructure with *N* server sites.

**Figure 2:**
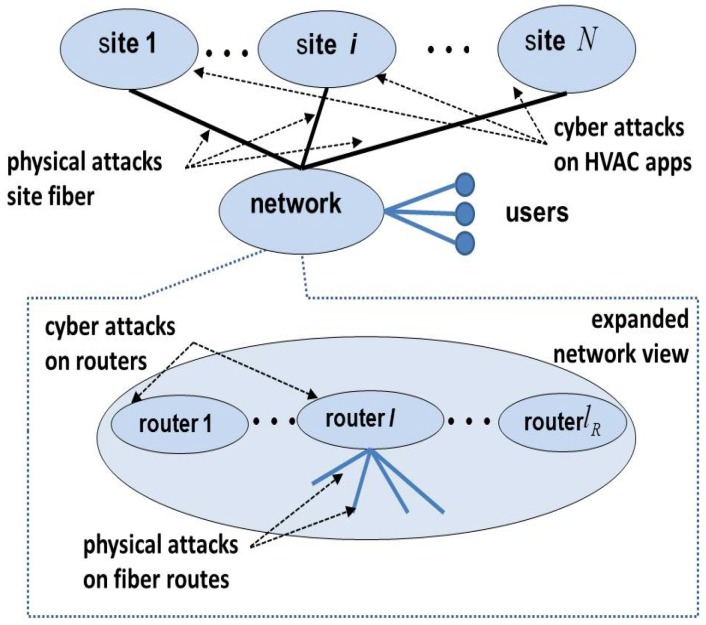
Network of a multi-site cloud server infrastructure.

A distributed cloud computing infrastructure consisting of *N* sites, each with $l_{i}$ servers at site *i*, i=1,2,…,N, has been studied by using separate cyber and physical models for each site in [2]. Here, we expand this model to incorporate both cyber and physical aspects of the HVAC of a site, namely its mobile phone app and cooling tower located outside the facility. The sites are connected over a wide-area network $S_{N+1}$, as shown in Figure 1. The components of the network include routers, each of which manages $l_{N+1}$ connections as shown in Figure 2.

This infrastructure is subject to a variety of cyber and physical attacks on its components. Cyber attacks on the servers may be launched remotely over the network since the servers are accessible to users. Meanwhile, routers are located at geographically-separated sites, and access to them is limited (to network administrators), so they are not as easily accessible over the network. Cyber attacks on routers require different techniques and represent different costs to the attacker compared to server attacks. Furthermore, this infrastructure is subject to physical attacks in the form of fiber cuts, which require a proximity access by the attacker. Cutting the network fibers that connect server sites to routers will disconnect the entire site, making it inaccessible to the users. Such attacks may also be launched on the network fibers between routers at different locations on the network.

The infrastructure provider may employ a number of reinforcements to protect against attacks, including replicating the servers and routers to support fail-over operations and installing physically-separated redundant fiber lines to the sites and between router locations. These measures could require significant costs and hence must be strategically chosen.

### 5.1. System-Level Correlations

**Figure 3:**
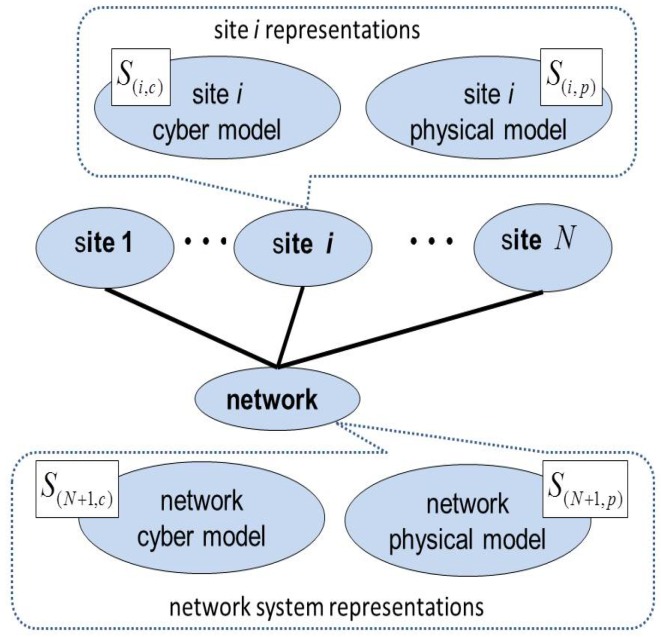
Representation of the cloud computing infrastructure.

The cyber and physical aspects of a site $S_{i}$ can be represented by using two finer sub-models $S_{\left(i,c\right)}$ and $S_{\left(i,p\right)}$ that correspond to the cyber and physical model, respectively. Similarly, those of the network $S_{N+1}$ are represented by $S_{\left(N+1,c\right)}$ and $S_{\left(N+1,p\right)}$, which are the cyber and physical models, respectively, as illustrated in Figure 3. Let $n_{\left(i,c\right)}$ and $n_{\left(i,p\right)}$ represent the cyber and physical components of $S_{i}$, which correspond to the number of components of $S_{\left(i,c\right)}$ and $S_{\left(i,p\right)}$, respectively, such that $n_{i}=n_{\left(i,c\right)}+n_{\left(i,p\right)}$. Let $x_{\left(i,c\right)}$ and $x_{\left(i,p\right)}$ denote the number of cyber and physical components that are reinforced, respectively, such that $x_{i}=x_{\left(i,c\right)}+x_{\left(i,p\right)}$. Similarly, $y_{\left(i,c\right)}$ and $y_{\left(i,p\right)}$ denote the number of cyber and physical components that are attacked, respectively, such that $y_{i}=y_{\left(i,c\right)}+y_{\left(i,p\right)}$. The relationships between these system-level models can be captured using refined versions of the aggregate correlation function as follows. For the wide-area network, we have:

$$C_{\left(N+1,c\right)}=l_{N+1}C_{\left(N+1,p\right)}$$

which reflects that a cyber attack on a router will disrupt all of its $l_{N+1}$ connections, thereby illustrating the amplification effect of these cyber attacks. For the server sites, we have a similar effect due to physical fiber attacks denoted by label $p_{f}$ reflected by:

$$C_{\left(i,p_{f}\right)}=l_{i}C_{\left(i,c\right)}$$

which indicates that at site $S_{i}$, the fiber disruption will disconnect all of its $l_{i}$ servers. Similarly, the cyber attack on the site’s HVAC app denoted by label $c_{h}$ leads to:

$$C_{\left(i,c_{h}\right)}=l_{i}C_{\left(i,c\right)}$$

which indicates that at site $S_{i}$, the HVAC disruption will affect all of its $l_{i}$ servers. In the limiting case, each component can be represented as a singleton sub-model $S_{i,j}$ such that $x_{i}=\sum_{j=1}^{n_{i}}x_{\left(i,j\right)}$ and $y_{i}=\sum_{j=1}^{n_{i}}y_{\left(i,j\right)}$. Here, $x_{\left(i,j\right)}\in \left\{0,1\right\}$ and $y_{\left(i,j\right)}\in \left\{0,1\right\}$ indicate if the component represented by $S_{i,j}$ is reinforced and attacked, respectively.

### 5.2. Component-Level Correlations

We now consider a special case where the attacker and provider choose the components of a constituent system to attack and reinforce, respectively, according to a uniform distribution. Corresponding to the site physical model $S_{\left(i,p\right)}$, i=1,2,…,N, there are ${\left[n_{\left(i,p\right)}-x_{\left(i,p\right)}\right]}_{+}$ non-reinforced fiber connections, where ${\left[x\right]}_{+}=x$ for x>0, and ${\left[x\right]}_{+}=0$ otherwise. Similarly, there are ${\left[n_{\left(i,c\right)}-x_{\left(i,c\right)}\right]}_{+}$ non-reinforced servers. If a cyber component (i.e., a server) is reinforced, it will survive a cyber attack, but can be brought down indirectly by a fiber attack. Then, the probability that a cyber-reinforced component survives $y_{\left(i,p\right)}$ fiber attacks is approximated by:

$$p_{(i,c)|R}=\frac{f_{\left(i,c\right)}}{1+l_{i}{\left[y_{\left(i,p\right)}-x_{\left(i,p\right)}\right]}_{+}}$$

where the normalization constant $f_{\left(i,c\right)}$ is appropriately chosen.

On the other hand, if a cyber component is not reinforced, it can be brought down by either a direct cyber attack or indirectly through a fiber attack. Thus, we approximate the survival probability of a cyber component at site *i* as:

$$p_{(i,c)|W}=\frac{f_{\left(i,c\right)}}{1+y_{\left(i,c\right)}+l_{i}{\left[y_{\left(i,p\right)}-x_{\left(i,p\right)}\right]}_{+}}$$

which reflects the additional lowering of the survival probability in inverse proportion to the level of cyber attack $y_{\left(i,c\right)}$. Under the independence of component attacks and reinforcements, the survival probability of the cyber sub-model $S_{\left(i,c\right)}$ is given by:

(4) $$P_{\left(i,c\right)}=p_{(i,c)|R}^{x_{\left(i,c\right)}}p_{(i,c)|W}^{n_{\left(i,c\right)}-x_{\left(i,c\right)}}$$

which in turn provides:

$$\frac{\partial P_{\left(i,c\right)}}{\partial x_{\left(i,c\right)}}=P_{\left(i,c\right)}\ln\left(\frac{p_{(i,c)|R}}{p_{(i,c)|W}}\right)$$

Using the above formulae, for cyber model $S_{\left(i,c\right)}$ of site $S_{i}$, we have:

$$\Lambda_{\left(i,c\right)}\left(x_{\left(i,p\right)},y_{\left(i,c\right)},y_{\left(i,p\right)}\right)=\ln\left(1+\frac{y_{\left(i,c\right)}}{1+l_{i}{\left[y_{\left(i,p\right)}-x_{\left(i,p\right)}\right]}_{+}}\right)$$

It is interesting to note that the system multiplier function $\Lambda_{\left(i,c\right)}$ does not depend on the cyber reinforcement term $x_{\left(i,c\right)}$ even though it corresponds to $\frac{\partial P_{\left(i,c\right)}}{\partial x_{\left(i,c\right)}}$. The function, however, depends on the physical reinforcement term $x_{\left(i,p\right)}$.

Under the statistical independence of cyber and physical attacks, for the cyber and physical sub-models, namely, $S_{\left(i,c\right)}$ and $S_{\left(i,p\right)}$, respectively, we have the following generalization of Equation (4):

$$\begin{array}{cc} P_{i}= & p_{(i,c)|R}^{x_{\left(i,c\right)}}p_{(i,c)|W}^{n_{\left(i,c\right)}-x_{\left(i,c\right)}}p_{(i,p)|R}^{x_{\left(i,p\right)}}p_{(i,p)|W}^{n_{\left(i,p\right)}-x_{\left(i,p\right)}} \end{array}$$

or equivalently:

$$\begin{array}{c} \ln P_{i}=n_{\left(i,c\right)}\ln p_{(i,c)|W}+x_{\left(i,c\right)}\ln\left(\frac{p_{(i,c)|R}}{p_{(i,c)|W}}\right)+n_{\left(i,p\right)}\ln p_{(i,p)|W}+x_{\left(i,p\right)}\ln\left(\frac{p_{(i,p)|R}}{p_{(i,p)|W}}\right) \end{array}$$

By differentiating the equation with $x_{\left(i,c\right)}$, we obtain:

$$\frac{\partial P_{i}}{\partial x_{\left(i,c\right)}}=\ln\left(\frac{p_{(i,c)|R}}{p_{(i,c)|W}}\right)P_{i}=\Lambda_{\left(i,c\right)}P_{i}$$

Then, by noting that $\frac{\partial x_{i}}{\partial x_{\left(i,c\right)}}=1$, we obtain:

$$\frac{\partial P_{i}}{\partial x_{i}}=\Lambda_{\left(i,c\right)}P_{i}$$

which enables us to approximate $\Lambda_{i}$ by $\Lambda_{\left(i,c\right)}$.

Consider that the HVAC sub-model $S_{\left(i,h\right)}$ of site *i* is further decomposed into cyber and physical singleton sub-models represented by $S_{\left(i,c_{h}\right)}$ and $S_{\left(i,p_{h}\right)}$, respectively. Then, we have:

(5) $$\Lambda_{\left(i,c_{h}\right)}=\ln\left(1+\frac{y_{\left(i,c_{h}\right)}}{1+l_{i}{\left[y_{\left(i,c_{h}\right)}-x_{\left(i,c_{h}\right)}\right]}_{+}}\right)$$

which corresponds to a cyber attack on and defense of the HVAC app. Similarly, we have:

$$\Lambda_{\left(i,p_{h}\right)}=\ln\left(1+\frac{y_{\left(i,p_{h}\right)}}{1+l_{i}{\left[y_{\left(i,p_{h}\right)}-x_{\left(i,p_{h}\right)}\right]}_{+}}\right)$$

which corresponds to a physical attack on and defense of the HVAC cooling tower.

### 5.3. Expected Capacity Estimates

We now consider the capacity of the infrastructure under $x_{i}$ reinforcements and $y_{i}$ attacks on components of $S_{i}$, which can be further partitioned into the corresponding values of sub-systems of $S_{i}$.

#### 5.3.1. Sum-Form and Product-Form

Based on the estimates from Section 4.3, for the expected capacity $N_{I}^{A}$ of the sub-models of $S_{i}$, the dependence on $y_{\left(i,c\right)}$ and ${\left[y_{\left(i,p\right)}-x_{\left(i,p\right)}\right]}_{+}$ is more direct, and it is qualitatively similar for both sum-form and product-form, since the term $\Lambda_{i}$ appears in the denominator. Then, we obtain the following expected capacity estimates: for the sum-form,

$$N_{I}^{+}=\sum_{i=1}^{N}\left(\frac{n_{i}\frac{\partial L_{D}}{\partial x_{i}}}{g_{D}\ln\left(1+\frac{y_{\left(i,c\right)}}{1+l_{i}{\left[y_{\left(i,p\right)}-x_{\left(i,p\right)}\right]}_{+}}\right)}\right)$$

and for the product form,

$$N_{I}^{\times }=\left(1-P_{I}\right)\sum_{i=1}^{N}\left(\frac{n_{i}\frac{\partial L_{D}}{\partial x_{i}}}{L_{D}\ln\left(1+\frac{y_{\left(i,c\right)}}{1+l_{i}{\left[y_{\left(i,p\right)}-x_{\left(i,p\right)}\right]}_{+}}\right)}\right)$$

In both cases, the multipliers $n_{i}$, $g_{D}$ and $L_{D}$ are positive, and it is reasonable to assume the condition $\frac{\partial L_{D}}{\partial x_{i}}\geq 0$, as described above. Thus, the expected capacity decreases with the number of cyber attacks $y_{\left(i,c\right)}$. The opposite trend is true with respect to ${\left[y_{\left(i,p\right)}-x_{\left(i,p\right)}\right]}_{+}$, which implies no effect if the number of reinforced components is at least as large as the number of component attacks, and otherwise, the expected capacity increases with the difference. In both cases, the dependence on the number of servers $l_{i}$ at site *i* is qualitatively similar in that the expected capacity increases proportional to its logarithm.

The term $\left(n_{i}\frac{\xi_{i}^{A}}{\Lambda_{i}}\right)$ that corresponds to site $S_{i}$ can be further refined by decomposing into its sub-models, which provides insight into their individual effects. The impact of the HVAC control app at site *i* is reflected in its corresponding term:

$$\frac{\xi_{i}^{A}}{\ln\left(1+\frac{y_{\left(i,c_{h}\right)}}{1+l_{i}{\left[y_{\left(i,c_{h}\right)}-x_{\left(i,c_{h}\right)}\right]}_{+}}\right)}$$

obtained from Equation (5), which shows that reinforcing the app, that is $x_{\left(i,c_{h}\right)}=1$, nullifies the amplification effect of $l_{i}$ since ${\left[y_{\left(i,c_{h}\right)}-x_{\left(i,c_{h}\right)}\right]}_{+}=0$ for both sum-form and product-form utility functions. Such an analysis can be carried out for other critical components of the sites to gain information on which components to reinforce for higher utility. In particular, reinforcing the site fiber routes will have a similar effect on nullification, but server reinforcements will have somewhat lesser impact.

#### 5.3.2. Composite Utility Functions

We now obtain the following expected number of servers for the composite utility functions,

$$N_{I}=\sum_{i=1}^{N}\left(-\frac{n_{i}F_{G,L}^{D,i}}{L_{G,L}^{D}\ln\left(1+\frac{y_{\left(i,c\right)}}{1+l_{i}{\left[y_{\left(i,p\right)}-x_{\left(i,p\right)}\right]}_{+}}\right)}\right)$$

In the equation, $n_{i}$ is positive, and it is reasonable to assume that $-\frac{F_{G,L}^{D,i}}{L_{G,L}^{D}}\geq 0$, since $\frac{\partial P_{I}}{\partial x_{i}}=-\frac{F_{G,L}^{D,i}}{L_{G,L}^{D}}$ at NE, and the survival probability of entire infrastructure $P_{I}$ does not decrease with $x_{i}$. Thus, the expected capacity decreases with $y_{\left(i,c\right)}$, and the opposite is true with respect to ${\left[y_{\left(i,p\right)}-x_{\left(i,p\right)}\right]}_{+}$, as discussed in the previous section. In both cases, the dependence on the number of servers $l_{i}$ at site *i* is qualitatively similar in that the expected capacity increases proportional to its logarithm, also as in the previous section. As in sum-form and product-form utility functions, the term:

$$\left(-\frac{n_{i}}{\Lambda_{i}}\frac{F_{G,L}^{D,i}}{L_{G,L}^{D}}\right)$$

can be decomposed using sub-models of site *i* to assess the impacts of its parts, in particular its components. For the HVAC app at site *i*, we have the corresponding term:

$$\left(-\frac{F_{G,L}^{D,(i,c_{h})}}{L_{G,L}^{D}\ln\left(1+\frac{y_{\left(i,c_{h}\right)}}{1+l_{i}{\left[y_{\left(i,p_{h}\right)}-x_{\left(i,p_{h}\right)}\right]}_{+}}\right)}\right)$$

which shows that reinforcing the HVAC app nullifies the amplification by factor $l_{i}$, even under the more general utility function since $l_{i}$ does not appear in the dependent term $F_{G,L}^{D,(i,c_{h})}$. Furthermore, such an analysis can be carried out for other components, and in a limiting case, each component can be modeled as a singleton sub-model, in which case their attack and reinforcement variables are Boolean.

The dependencies considered here for the sub-models are quite simple as a result of the statistical independence and uniform distributions of reinforcements and attacks. Even under such simple conditions, the detailed NE conditions are quite complex to characterize, but they do provide qualitative insights into the effects of underlying parameters.

## 6. Conclusions

We consider a class of infrastructures with multiple systems, wherein the communications network plays an asymmetric role by providing the critical connectivity between them. By utilizing correlations at the system- and component-level, we formulated the problem of ensuring the infrastructure survival as a game between an attacker and a provider, by using composite utility functions that generalize the sum-form and product-form utility functions. We derived Nash equilibrium conditions in terms of composite gain-cost and composite multiplier, which provide compact expressions for individual system survival probabilities and also the expected number of operational components. This paper presented a unified account of partial results that were separately developed for: sum-form utility functions [5] and under asymmetric network conditions [1]; product-form utility functions [8]; composite utility functions [2]; composite utility functions under asymmetric network conditions [3]; and detailed derivations for multi-site cloud server infrastructure [4]. These results extend previous results on interconnected systems [30,32] and cyber-physical infrastructures [31] by using the composite utility functions. We presented a comprehensive treatment of the three utility functions, including more illustrative details of the sum-form and product-form utility functions. For multi-site cloud infrastructures, we explicitly related the correlation functions and system multiplier functions to the infrastructure parameters, which in turn provided us insights into the estimates for system survival probabilities and the expected capacity. In particular, by employing sub-models of the sites, the effect of parts of the system on the expected capacity could be inferred by using the corresponding multiplier functions.

The formulation studied in this paper can be extended to include cases where targeted attacks and reinforcements of specific individual components are explicitly represented. The system models here incorporate the same level of detail in that they all consist of components, and it would be of future interest to incorporate varying levels of detail in them, for example by replacing components with the recursively-defined systems. The utility functions considered in this paper do not explicitly use the capacity term. Instead, they are driven by the infrastructure level considerations by using $P_{I}$, which in turn leads to expressions for the capacity that involve other terms that contribute to $P_{I}$. It is of future interest to compare this formulation to ones whose utility functions contain the expected capacity term in place of infrastructure survival probability terms. Another future direction is to consider the simultaneous cyber and physical attacks on multiple systems and components and sequential game formulations of this problem. Performance studies of our approach using more detailed models of cloud computing infrastructure, smart energy grid infrastructures and high-performance computing complexes would be of future interest. 

## Figures and Tables

**Table 1:** Gain and cost terms and their multipliers for sum-form and product-form utilities of the provider.

	$F_{D,G}$	$G_{D}$	$F_{D,L}$	$L_{D}$
sum-form: $U_{D+}$	$\left[1-P_{I}\right]$	$g_{D}$	1	$L_{D}$
product-form: $U_{D\times }$	0	0	$\left[1-P_{I}\right]$	$L_{D}$

**Table 2:** Gain and cost terms, their multipliers and other terms for sum-form and product-form utilities of the provider.

	$F_{D,G}$	$G_{D}$	$F_{D,L}$	$L_{D}$	$\frac{\partial F_{D,G}}{\partial P_{I}}$	$\frac{\partial G_{D}}{\partial x_{i}}$	$\frac{\partial F_{D,L}}{\partial P_{I}}$	$L_{G,L}^{D}$	$F_{G,L}^{D,i}$
sum-form: $U_{D+}$	$\left[1-P_{I}\right]$	$g_{D}$	1	$L_{D}$	−1	0	0	$-g_{D}$	$\frac{\partial L_{D}}{\partial x_{i}}$
product-form: $U_{D\times }$	0	0	$\left[1-P_{I}\right]$	$L_{D}$	0	0	−1	$-L_{D}$	$\left[1-P_{I}\right]\frac{\partial L_{D}}{\partial x_{i}}$
